# Burden of head and neck cancers in five East Asian countries from 1990 to 2023: Observation, comparison, and forecast from the global burden of disease study 2023

**DOI:** 10.1371/journal.pone.0349297

**Published:** 2026-05-15

**Authors:** Yinghong Li, Mingjie Tang, Shiwei Li, Peipei Yang, Qiurong Li, Peng Shu

**Affiliations:** 1 Affiliated Hospital of Nanjing University of Chinese Medicine, Nanjing, China; 2 Nanjing University of Chinese Medicine, Nanjing, China; 3 Jiangsu Provincial Hospital of Chinese Medicine, Nanjing, China; 4 Central South University, Changsha, China; National Center for Chronic and Noncommunicable Disease Control and Prevention, Chinese Center for Disease Control and Prevention, CHINA

## Abstract

**Background:**

Head and neck cancer (HNC) poses a significant public health challenge worldwide, yet the long-term trends and heterogeneity of its burden within East Asia remain inadequately characterized. This study aims to provide a comprehensive assessment of the HNC burden in five East Asian countries from 1990 to 2023 and project future trends.

**Methods:**

Utilizing data from the Global Burden of Disease (GBD) 2023, we analyzed the incidence, prevalence, mortality, and disability-adjusted life years (DALYs) of HNC in China, Japan, the Republic of Korea, the Democratic People’s Republic (DPR) of Korea, and Mongolia. We employed a comprehensive analytical approach encompassing age-standardized rates, temporal trends, Joinpoint regression, risk factor attribution, age-period-cohort analysis, as well as decomposition and forecasting analyses.

**Results:**

From 1990 to 2023, substantial heterogeneity was observed. China demonstrated significant declines in age-standardized incidence rate (ASIR) and mortality rate (ASMR). Conversely, Japan experienced concerning increases in ASIR and ASMR. The Republic of Korea maintained a stable ASIR while achieving a marked ASMR reduction. The Democratic People’s Republic of Korea showed increases in both ASIR and ASMR, while Mongolia reported declines in ASIR and ASMR. Age distribution shifted markedly towards older populations in China, Japan, and the Republic of Korea. Smoking remained the predominant risk factor across HNC subtypes. Forecasts to 2038 project a continued rise in ASIR for Japan and the DPR of Korea, and a high ASMR for the DPR of Korea.

**Conclusion:**

The HNC burden in East Asia exhibits divergent national trajectories, with smoking remaining the predominant attributable risk factor. Further research is needed to elucidate underlying causes of these cross-country disparities.

## Introduction

Head and neck cancer (HNC) encompasses a heterogeneous group of malignancies originating primarily from the mucosal epithelium of the upper aerodigestive tract, including sites such as the oral cavity, oropharynx, hypopharynx, larynx, and nasal cavity [1]. Although HNC does not rank among the top five cancers in terms of global burden, it nevertheless represents a substantial public health challenge, with approximately 1.71 million new cases and over 500,000 deaths reported worldwide in 2022 [2]. The development of HNC is strongly associated with several modifiable risk factors, notably tobacco use in both smoked and smokeless forms, excessive alcohol consumption, human papillomavirus (HPV) infection particularly in oropharyngeal carcinoma, and region-specific habits such as betel quid chewing [3–5]. Despite multimodal treatment approaches combining surgery, radiotherapy, and chemotherapy, the prognosis of HNC remains poor [6]. Surgical management of HNC often leads to functional and aesthetic impairments, including dysphagia, craniofacial deformity, and chronic pain, which significantly compromise patients’ quality of life and social engagement [7,8]. Moreover, compared to more common cancers such as lung and breast cancer, public awareness of HNC and early detection through screening are considerably underdeveloped [9]. Consequently, a large proportion of patients are diagnosed at locally advanced or metastatic stages, adversely affecting treatment response and long-term survival outcomes.

According to the Global Burden of Disease (GBD) 2021, an estimated 0.91 million new HNC cases occurred globally in 2021, reflecting a significant increase since 1990 [10]. East Asia, including China, Japan, the Republic of Korea, the Democratic People’s Republic (DPR) of Korea, and Mongolia, contributes substantially to the global HNC burden. The region’s considerable population size, rapid demographic aging, historically high tobacco consumption, prevalent alcohol use culture, and in certain areas such as mainland China, betel quid chewing habits, collectively contribute to a distinct HNC risk profile [11]. In China, there are approximately 128,000 new HNC cases and 65,000 related deaths annually, with an upward trend observed in recent years [12]. Furthermore, the incidence of HPV-associated oropharyngeal cancer has been increasing in several developed countries within the region, such as Japan and the Republic of Korea [4,13]. In contrast, countries like Mongolia and the DPR of Korea, constrained by limited healthcare resources, have scarcely systematically assessed the true burden of HNC. Despite the considerable regional burden, there remains a lack of systematic comparative analyses of the disease burden and long-term trends of HNC across these five East Asian countries based on the most recent data. Existing studies are largely confined to single nations or specific cancer subtypes, which limits comprehensive insight into shared regional patterns, cross-country variations, and the impacts of socioeconomic development and lifestyle changes over the past three decades.

Therefore, this study aimed to utilize latest GBD 2023 to comprehensively evaluate the incidence, prevalence, mortality, and disability-adjusted life years (DALYs) burden of HNC and their temporal trends from 1990 to 2023 across five East Asian countries. We also conducted Joinpoint regression analysis, decomposition analysis, risk factor analysis, and forecasting analysis to provide critical evidence for developing tailored regional prevention and control strategies.

## Methods

### Data source and collection

GBD 2023 is an international research initiative that provides publicly available burden estimates for 375 diseases and 88 risk factors across 204 countries and territories [14,15]. The data are accessible through the Global Health Data Exchange (https://vizhub.healthdata.org/gbd-results/). The GBD study employs a standardized methodological framework for data identification, extraction, and processing, with detailed descriptions of its principles and procedures available in prior publications [16–18]. This analysis focused on HNC, which includes cancers of the lip and oral cavity (LOC), nasopharynx (NPC), larynx (LC), and other pharynx (OPC). The corresponding International Classification of Diseases (ICD) codes are provided in S1 Table. We systematically evaluated and compared the burden of HNC from 1990 to 2023 across five East Asian countries: China, Japan, the Republic of Korea, the DPR of Korea, and Mongolia. The primary outcome measures were incidence, prevalence, mortality, and DALYs, presented as both absolute numbers and age-standardized rates (ASRs). DALYs, a composite indicator of fatal and non-fatal health losses, are computed as the sum of Years of Life Lost (YLLs) and Years Lived with Disability (YLDs). To quantify uncertainty in the modeling and sampling processes, 1000 posterior draws were generated. The reported 95% uncertainty intervals (UIs) represent the 2.5th and 97.5th percentiles of the ordered draws.

### Temporal trend analysis

To quantify temporal trends, we calculated the estimated annual percentage change (EAPC) in the ASRs, along with its 95% confidence interval (CI), using a generalized linear regression model [19]. A trend was considered increasing if both the EAPC and its 95% CI were greater than zero, and decreasing if both were less than zero. A trend was deemed stable if the 95% CI included zero.

### Joinpoint Regression Analysis

To identify significant turning points in the trends of HNC burden, we performed Joinpoint regression analysis, a method widely used in epidemiological studies [20]. This analysis was applied to assess the dynamic trends of the age-standardized incidence rate (ASIR), prevalence rate (ASPR), mortality rate (ASMR), and DALYs rate (ASDALYR) for HNC from 1990 to 2023 across the five East Asian countries. We calculated the annual percentage change (APC) and its 95% confidence interval (CI) for each segment identified between joinpoints. To provide an overall summary measure of the trend over the entire period, the average annual percentage change (AAPC) and its 95% CI were also computed. A statistically significant increasing trend was indicated if both the APC/AAPC and the lower limit of its 95% CI were greater than 0. Conversely, a significant decreasing trend was indicated if both the APC/AAPC and the upper limit of its 95% CI were less than 0.

### Age-period-cohort analysis

The age-period-cohort model was employed to estimate the independent effects of age, period, and cohort on disease incidence. Within this framework, the age effect reflects changes in HNC risk associated with aging, the period effect captures temporal variations influencing outcomes across all age groups, and the cohort effect represents risk differences among birth cohorts attributable to evolving early-life exposures or changing birth characteristics [21]. To thoroughly investigate temporal trends and potential stratified factors in HNC incidence, this study applied the age-period-cohort model to systematically assess the burden of HNC incidence across five East Asian countries.

Incidence and corresponding population data were integrated into a consistent analytical structure, with all measures averaged over five-year intervals. To balance model complexity with smoothed temporal trends [22], data from GBD 2023 were stratified into five-year age groups (<5, 5–9, 10–14, …, ≥ 95 years) and six consecutive five-year calendar periods (1994–1998, 1999–2003, 2004–2008, 2009–2013, 2014–2018, and 2019–2023). Birth cohorts were derived by subtracting the age from the period. The significance of estimated parameters was evaluated using the Wald chi-squared test. Parameter estimation for the age-period-cohort analysis was conducted using the tool developed by the National Institutes of Health (NIH) (https://analysistools.cancer.gov/apc/) [23].

### Association between ASRs and the Socio-demographic Index (SDI)

The SDI is a composite indicator of development status, strongly correlated with health outcomes [24]. It is calculated based on the geometric mean of three metrics: lag-distributed income per capita, average years of education among individuals aged 25 and older, and the total fertility rate. SDI values range from 0 to 1 and are categorized as follows: high (>0.81), high-middle (0.71–0.81), middle (0.62–0.71), low-middle (0.47–0.62), and low (<0.47), with higher values reflecting greater socioeconomic development. We employed Spearman correlation analysis to examine the association between the ASRs of HNC and the SDI across the five East Asian countries.

### Decomposition Analysis

We conducted a decomposition analysis to partition the total change in HNC DALYs over the study period into contributions from specific demographic and epidemiological factors [25]. Following an established methodology, the change was attributed to three primary components: population growth, population aging, and epidemiological change. This approach allows for a multidimensional understanding of how the overall trend in HNC burden is shaped by the interplay of epidemiological shifts and demographic dynamics.

### Risk Factor Attribution

The contribution of risk factors to the HNC DALY burden was assessed using population attributable fractions (PAFs) from GBD 2023. As risk factors for HNC cannot be validly pooled across its anatomical subtypes, the analysis was performed separately for LOC, NPC, LC and OPC. The risk factors considered for these subtypes included smoking, chewing tobacco, high alcohol use, and occupational exposures to formaldehyde, sulfuric acid, and asbestos. The methodological details for calculating PAFs have been described in previous GBD publications [26]. This study assessed the percentage contribution of these risk factors to DALYs in 2023 in five East Asian countries.

### Forecasting Analysis

We employed the Autoregressive Integrated Moving Average (ARIMA) model to forecast future trends in HNC burden. The ARIMA model is a classical time-series forecasting method that identifies and models autocorrelation, trend, and seasonality through its components of autoregression, differencing, and moving average. This established approach is well-suited for predicting future trends based on historical GBD data [27,28].

### Ethical considerations

This study utilized publicly available data from GBD 2023 and did not use any individual-level data. Thus, no ethics approval was needed.

### Statistical analysis

All statistical analyses were performed using R (version 4.4.1). A two-sided p-value of less than 0.05 was considered statistically significant.

## Results

### Overall trends in HNC burden in the five East Asian countries, 1990–2023

From 1990 to 2023, the burden of HNC exhibited a distinct pattern across the five East Asian countries. Overall, incident cases, prevalent cases, deaths, and DALYs increased in all five countries. China recorded the largest absolute increases, with incident and prevalent cases rising by 97.21% and 173.70%, respectively. Nevertheless, its ASIR declined from 9.96 to 8.55 per 100 000, translating to a 14.16% decrease. It is noteworthy that China experienced the most substantial declines in ASMR and ASDALYR among the five countries, with reductions of 56.20% and 58.80%, respectively. In contrast, Japan not only saw its ASIR rise from 5.45 to 8.13 per 100,000, with an increase of 49.17%, but also recorded a 31.74% increase in ASMR, representing the largest percentage rise among the five nations. The Republic of Korea observed a moderate increase in ASIR by 11.93%, while its ASMR and ASDALYR declined by 43.75% and 48.46%, respectively. The DPR of Korea exhibited increases in both ASIR and ASMR, rising by 25.00% and 5.67%. Mongolia reported decreases in ASIR and ASMR by 35.40% and 41.70%. Among the five East Asian countries, China had the highest ASIR in both 1990 and 2023, while Japan’s figures were slightly elevated, and Mongolia consistently maintained relatively low levels. China also recorded the highest ASPR in both 1990 and 2023, whereas Mongolia had the lowest. Although China’s ASMR was the highest in 1990, it decreased to a relatively moderate level by 2023, at which point the DPR of Korea showed the highest ASMR (Table 1).

**Table 1:** Head and neck cancers in five East Asian countries: all-ages cases, age-standardized rates, and trends from 1990 to 2023.

Location	Measure	1990		2023		EAPC,1990–2023 (95% CI)
		All-ages cases (95% UI)	Age-standardized rates per 100,000 (95% UI)	All-ages cases (95% UI)	Age-standardized rates per 100,000 (95% UI)	
China	Incidence	94,277 (73,728–118,251)	9.96 (7.84 to 12.40)	185,921 (140,268–244,762)	8.55 (6.42 to 11.21)	−0.81 (−1.11 to −0.50)
	Prevalence	347,390 (258,861–464,873)	34.71 (26.23 to 46.30)	950,951 (689,458–1,319,740)	44.78 (32.19 to 62.02)	0.52 (0.21 to 0.83)
	Mortality	67,169 (52,773–80,420)	7.49 (5.90 to 9.03)	74,863 (62,356–90,159)	3.28 (2.73 to 3.94)	−2.90 (−3.13 to −2.68)
	DALYs	2,213,841 (1,715,993–2,642,049)	224.28 (175.91 to 268.08)	2,053,652 (1,726,426–2,458,513)	92.5 (77.91 to 109.99)	−3.16 (−3.40 to −2.91)
Japan	Incidence	9,165 (7,677–10,926)	5.45 (4.56 to 6.50)	26,729 (21,692–31,397)	8.13 (6.72 to 9.49)	0.84 (0.56 to 1.13)
	Prevalence	42,981 (34,483–52,826)	25.54 (20.44 to 31.43)	105,214 (82,714–128,042)	36.03 (28.52 to 43.9)	0.79 (0.48 to 1.09)
	Mortality	3,839 (3,373–4,329)	2.3 (2.02 to 2.60)	12,234 (10,097–14,176)	3.03 (2.61 to 3.47)	0.31 (0.07 to 0.55)
	DALYs	99,459 (87,374–112,548)	59.17 (51.95 to 66.95)	218,331 (187,681–250,477)	71.13 (62.57 to 81.16)	−0.01 (−0.28 to 0.27)
The Republic of Korea	Incidence	2,015 (1,302–2,738)	6.37 (4.22 to 8.54)	7,023 (4,565–11,675)	7.13 (4.66 to 11.70)	0.11 (−0.10 to 0.32)
	Prevalence	8,709 (5,237–12,344)	26.30 (16.26 to 36.82)	36,315 (22,363–62,946)	37.15 (23.17 to 63.37)	0.89 (0.64 to 1.14)
	Mortality	1,218 (836–1,578)	4.16 (2.94 to 5.35)	2,352 (1,763–3,387)	2.34 (1.76 to 3.34)	−2.25 (−2.46 to −2.04)
	DALYs	35,930 (24319–46758)	107.23 (73.25 to 138.95)	53,622 (40,535–77,146)	55.26 (42.16 to 78.31)	−2.40 (−2.56 to −2.24)
The Democratic People’s Republic of Korea	Incidence	927 (663–1319)	5.28 (3.77 to 7.49)	2,274 (1,563–3,311)	6.60 (4.54 to 9.57)	0.62 (0.44 to 0.79)
	Prevalence	3,488 (2,418–4,866)	18.74 (13.07 to 26.07)	9,013 (6,110–13,277)	25.94 (17.70 to 38.16)	0.99 (0.77 to 1.21)
	Mortality	643 (462–912)	3.88 (2.79 to 5.48)	1,393 (966–2,040)	4.10 (2.86 to 5.98)	0.07 (−0.06 to 0.20)
	DALYs	21,200 (15,112–30,300)	113.86 (81.91 to 161.55)	41,554 (28,545–61,041)	118.69 (81.95 to 173.65)	0.02 (−0.10 to 0.13)
Mongolia	Incidence	78 (53–106)	6.78 (4.62 to 9.22)	126 (93–170)	4.38 (3.25 to 5.93)	−1.93 (−2.16 to −1.70)
	Prevalence	227 (150–322)	18.22 (12.20 to 25.64)	418 (307–573)	13.82 (10.14 to 18.96)	−1.34 (−1.54 to −1.14)
	Mortality	60 (41–82)	5.42 (3.69 to 7.49)	86 (64–115)	3.16 (2.34 to 4.24)	−2.26 (−2.49 to −2.04)
	DALYs	1,911 (1,286–2,640)	155.55 (104.97 to 214.63)	2,830 (2,109–3,757)	92.37 (68.72 to 123.29)	−2.23 (−2.44 to −2.02)

All EAPC values were derived from the age‑standardized incidence rate, prevalence rate, mortality rate, and disability-adjusted life years rate, respectively.

EAPC, estimated annual percentage change; CI, confidence interval; DALYs, disability-adjusted life years; UI, uncertainty interval.

There was substantial heterogeneity in the DALYs burden across different subtypes of HNC among the five East Asian countries. China demonstrated the heaviest absolute burden for LOC, with DALYs increasing from 347,066 in 1990–543,250 in 2023, representing a 56.52% rise. Mongolia and Japan experienced the most pronounced percentage changes in DALYs, with increases of 137.80% and 128.50%, respectively. For NPC, China carried the highest absolute burden; however, it was the only country to exhibit a decline in DALYs, with a reduction of 38.04%. In contrast, Japan recorded the greatest increase in NPC DALYs, which rose to 22,509, reflecting a 73.66% growth. Regarding NC, although China continued to bear the heaviest absolute burden, Mongolia showed the most marked percentage change, with an increase of 166.84%. For OPC, Japan and Republic of Korea had the largest relative rises in DALYs, at 279.68% and 249.13%, respectively (S2 Table and S1 Fig).

### Age and sex patterns of HNC in five East Asian countries

**Fig 1:**
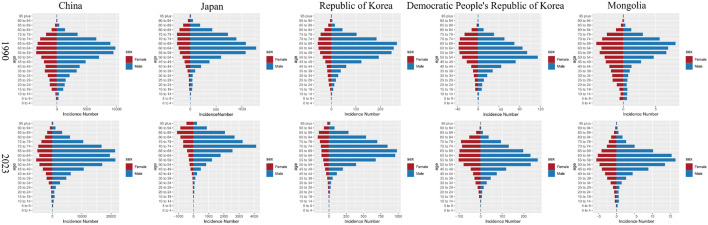
Number of head and neck cancer incident cases across different age groups in five East Asian countries in 1990 and 2023.

A comparative age- and sex-stratified analysis of the five East Asian countries between 1990 and 2023 revealed pronounced heterogeneity in their respective age distribution patterns. Notably, China and Japan exhibited distinct characteristics of population aging, with the peak age groups for both incident and mortality cases shifting demonstrably towards older populations by 2023, indicating a concentrating disease burden among the elderly. The Republic of Korea demonstrated a similar, albeit more moderate, aging trend. In contrast, the DPR of Korea maintained a disease burden predominantly concentrated in young and middle-aged adults, showing relatively limited change compared to 1990. In Mongolia, while the incidence peak in 2023 remained within young and middle-aged groups, there was a relative increase in the proportion of mortality among the elderly, suggesting early signs of an epidemiological transition. Overall, the burden of both incidence and mortality was consistently and significantly higher in males than in females across all age groups, a disparity persistently observed in all countries studied (Fig 1 and S2 Fig).

**Fig 2:**
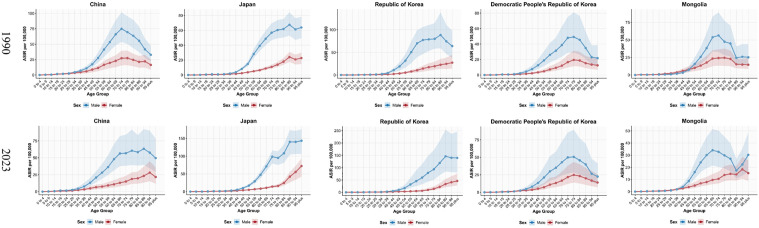
Comparison of ASIR for different age groups in five East Asian countries in 1990 and 2023. ASIR, age-standardized incidence rate.

Regarding age-specific incidence, China demonstrated a marked age-shift over this period. The peak age-specific incidence was observed in the 70–74 years age group for both sexes in 1990, shifting to the 85–89 years group in males and the 90–94 years group in females by 2023. In contrast, Japan, starting from an older baseline of 85–89 years in 1990, progressed to the 95 + age group for both sexes in 2023. The Republic of Korea exhibited a stable pattern, with peak age-specific incidence consistently at 85–89 years for males and 95+ for females. Conversely, the DPR of Korea maintained the youngest peak, at 75–79 years. Mongolia presented a divergent pattern, where the peak age-specific incidence for males shifted younger from 70–74–65–69 years, while that for females shifted significantly older, from 75–79–90–94 years. A similar progression toward older peak ages was observed for age-specific mortality. In China, the peak age-specific mortality shifted from 80–84 years (males) and 90–94 years (females) in 1990 to ≥ 95 years for both sexes in 2023. Japan consistently reported the oldest peak mortality at 95 + . In the Republic of Korea, the peak age for males advanced from 85–89–95 + , while remaining at 95+ for females. The DPR of Korea showed no change, with peaks at 80–84 (males) and 95+ (females). Finally, Mongolia’s peak mortality age for males shifted dramatically from 70–74–95 + , while remaining at 95+ for females. Collectively, these findings indicate a pronounced trend of aging in HNC burden in China, Japan, and the Republic of Korea. The stable or younger peaks observed in the DPR of Korea and for Mongolian males, however, suggest a different epidemiological profile (Fig 2 and S3 Fig).

### Temporal trends

**Fig 3:**
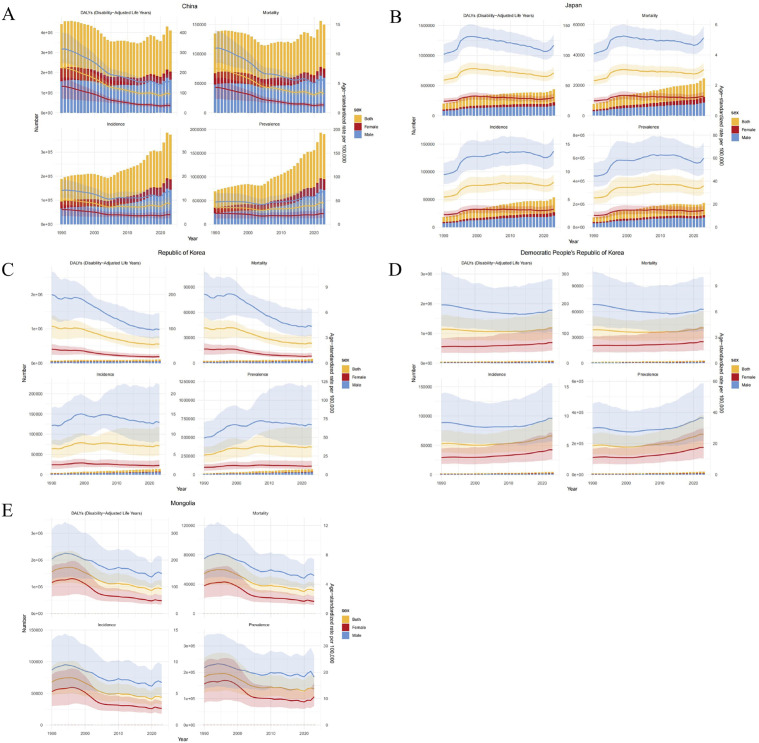
The trends in numbers and rates of incidence, prevalence, mortality, and DALYs for head and neck cancer in five East Asian countries from 1990 to 2023. **A.** China, **B.** Japan, **C.** Republic of Korea, **D.** Democratic People’s Republic of Korea, **E.** Mongolia; DALYs, disability-adjusted life years.

China exhibited the heaviest overall HNC burden; however, from 1990 to 2023, its ASIR, ASMR, and ASDALYR decreased substantially, indicating a gradual alleviation of the burden. Notably, Japan demonstrated an upward trend in ASIR, ASPR, and ASMR for HNC during the same period, suggesting a progressively worsening burden. In the Republic of Korea, the ASIR for HNC remained stable, while ASMR and ASDALYR showed significant declines. The DPR of Korea exhibited a slight increasing trend in ASIR, with ASMR and ASDALYR remaining stable. Mongolia experienced a substantial reduction in its HNC burden, with all indicators demonstrating declining trends. Furthermore, we observed that across all five countries, the HNC burden was consistently higher in males than in females (Fig 3 and Table 1).

### Joinpoint Regression Analysis

**Fig 4:**
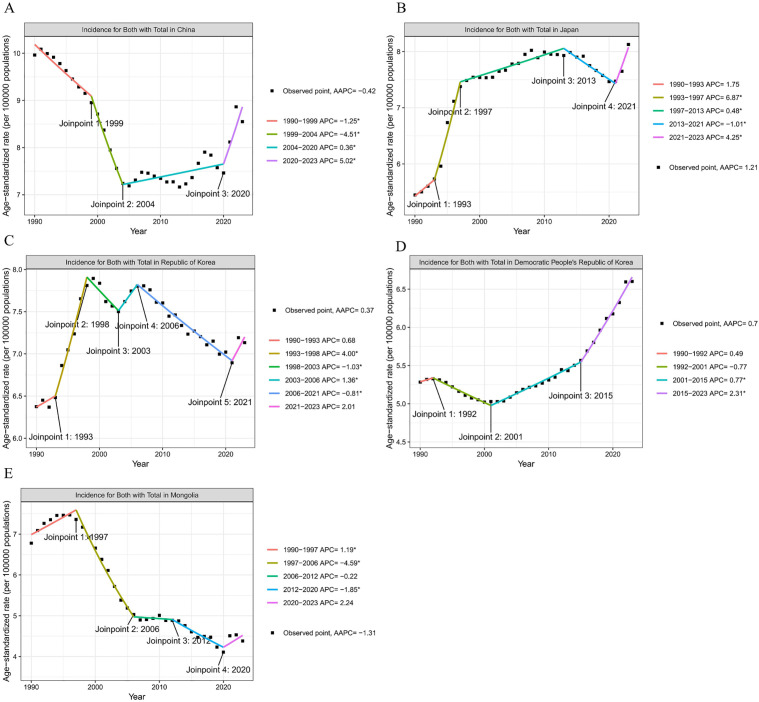
Joinpoint regression analysis of ASIR of head and neck cancer in five East Asian countries from 1990 to 2023. **A.** China, **B.** Japan, **C.** Republic of Korea, **D.** Democratic People’s Republic of Korea, **E.** Mongolia; ASIR, age-standardized incidence rate; *indicates a *P*-value less than 0.05.

The ASIR and ASMR of HNC in East Asian countries exhibited distinct temporal patterns. In China, the ASIR demonstrated a consistent downward trend (AAPC = −0.42, 95% CI: −0.56 to −0.28), with marked declines observed during specific periods (1990–1999, APC = −1.25 [95% CI: −1.71 to −0.55]; 1999–2004, APC = −4.51 [95% CI: −6.64 to −3.32]), followed by a recent increase (2020–2023, APC = 5.02 [95% CI: 2.74 to 8.61]). Japan experienced a significant overall increase in ASIR (AAPC = 1.21, 95% CI: 1.12 to 1.30), characterized by an initial rapid rise (1993–1997, APC = 6.87 [95% CI: 6.15 to 7.78]), a subsequent period of much slower growth (1997–2013, APC = 0.48 [95% CI: 0.36 to 0.62]), a slight decline (2013–2021, APC = −1.01 [95% CI: −1.58 to −0.71]), and a final upward turn (2021–2023, APC = 4.25 [95% CI: 2.12 to 5.68]). The Republic of Korea showed a slight overall upward trend in ASIR (AAPC = 0.37, 95% CI: 0.29 to 0.44). The DPR of Korea exhibited a mild increasing trend of ASIR (AAPC = 0.70, 95% CI: 0.65 to 0.75). In contrast, Mongolia recorded the most pronounced decrease in ASIR (AAPC = −1.31, 95% CI: −1.46 to −1.18) (Fig 4 and S3 Table).

Regarding ASMR, China exhibited the fastest overall decline (AAPC = −2.42, 95% CI: −2.54 to −2.30). Japan experienced the most severe increase in mortality (AAPC = 0.83, 95% CI: 0.73 to 0.93). Both the Republic of Korea and Mongolia demonstrated clear downward trends of ASMR (AAPC = −1.73, 95% CI: −1.81 to −1.61; AAPC = −1.57, 95% CI: −1.70 to −1.43, respectively). Conversely, a slight upward trend of ASMR was observed in the DPR of Korea (AAPC = 0.14, 95% CI: 0.11 to 0.17) (S4 Fig, S3 Table).

### Age-period-cohort analysis

**Fig 5:**
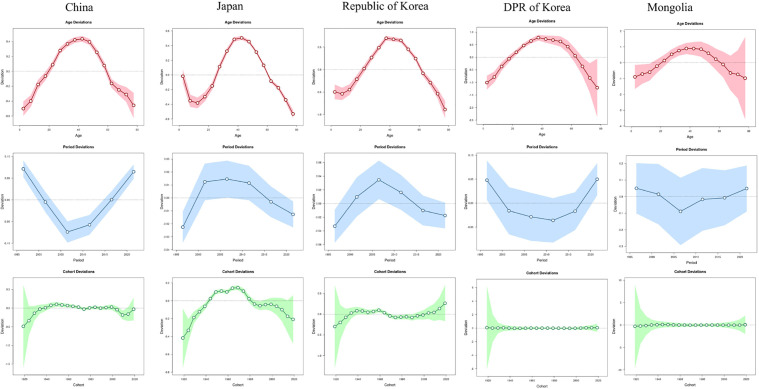
Age, period and cohort effects on head and neck cancer incidence in five East Asian countries. DPR of Korea, Democratic People’s Republic of Korea.

The age-period-cohort analysis revealed a consistent age-effect pattern across the five East Asian countries, with the risk of HNC incidence gradually increasing until approximately 40 years of age, after which it progressively declined. The peak incidence rate occurred within the 40–44 year age group in all countries studied. Period effects exhibited significant cross-national heterogeneity. China and the DPR of Korea demonstrated a U‑shaped temporal trend, characterized by an initial decline followed by a subsequent rise. In contrast, Japan and the Republic of Korea displayed an inverted U‑shaped period pattern, with incidence peaking in the middle periods. For Mongolia, the period effect had no significant impact on the incidence rate. Cohort effects further revealed distinct generational patterns. China showed minimal cohort-related fluctuations, suggesting limited birth‑cohort heterogeneity in HNC incidence. Conversely, Japan exhibited an inverted U‑shaped cohort trend, with the highest risk observed among mid‑range birth cohorts, while the Republic of Korea demonstrated pronounced cohort‑driven variability. For the DPR of Korea and Mongolia, the cohort effect was not statistically significant, implying that birth cohort did not substantially influence incidence trends in these populations (Fig 5).

### The association between ASRs and SDI

The analysis revealed a positive correlation between ASIR and ASPR of HNC and the SDI across the five East Asian countries. Particularly when the SDI exceeded 0.6, both ASIR and ASPR showed a gradual increase with rising SDI, suggesting that socioeconomic development may be associated with an increased risk of disease occurrence. In contrast, both ASMR and ASDALYR demonstrated a significant negative correlation with SDI within the region, indicating that higher SDI levels correspond to lower mortality and overall disease burden. Furthermore, the observed burden in China substantially exceeded the level predicted by its SDI, while Mongolia’s burden remained below the expected value (S5 Fig)

### Decomposition analysis

The decomposition analysis revealed distinct drivers behind the changes in HNC-related DALYs across the five East Asian countries from 1990 to 2023. In China, the overall DALYs decreased substantially. This reduction was overwhelmingly attributable to a strong, favorable epidemiological change (−1235.37%), which more than offset the combined increases linked to population growth (348.65%) and aging (786.72%). Conversely, Japan and the DRR of Korea experienced overall increases in DALYs. In Japan, population aging was the predominant driver, accounting for 65.37% of the total increase, followed by epidemiological change (31.01%). In the DPR of Korea, population aging (49.98%) and population growth (43.53%) were the major contributors. The Republic of Korea also saw an overall rise in DALYs, primarily fueled by population aging (234.52%). However, this was significantly counterbalanced by a substantial favorable epidemiological change (−189.12%). Mongolia recorded a minimal increase in DALYs. In summary, population aging consistently exerted upward pressure on HNC DALYs across all five nations. However, the trend in each country was decisively shaped by the magnitude and direction of its unique epidemiological change (S6 Fig and S4 Table).

### Risk factor contributions in 2023

**Fig 6:**
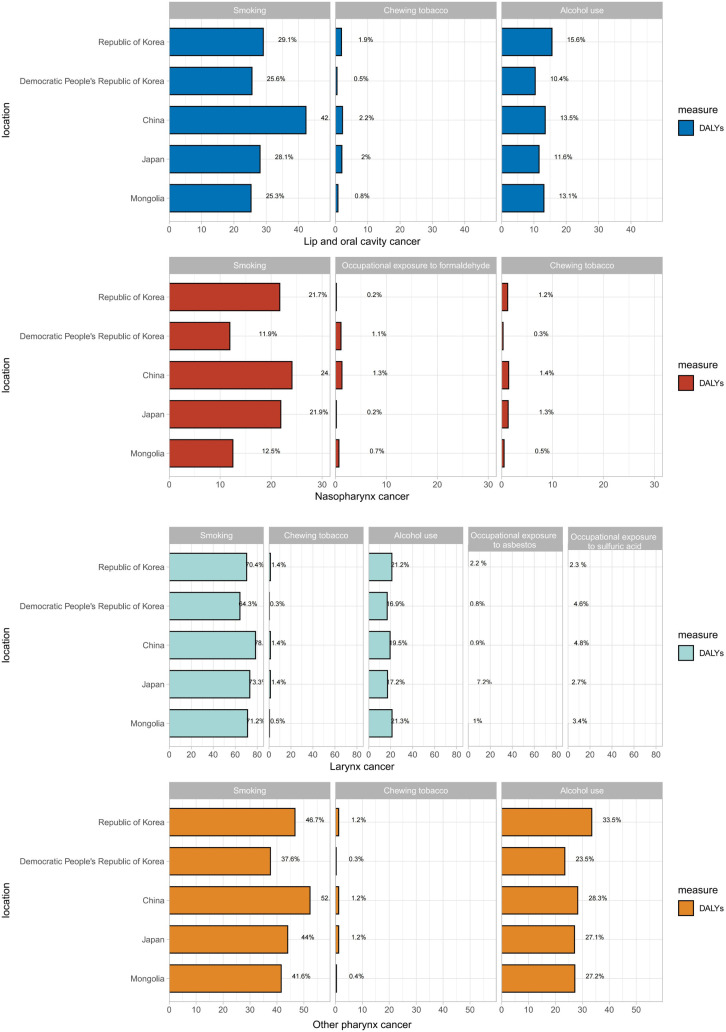
Risk factor attribution for DALYs of head and neck cancer subtypes in five East Asian countries in 2023. DALYs, disability-adjusted life years.

In 2023, the DALYs attributable to LOC across the five East Asian countries were predominantly driven by smoking, high alcohol use, and chewing tobacco. This pattern was most pronounced in China, where these risk factors accounted for 42.00%, 13.50%, and 2.20% of the burden, respectively. For NPC, smoking was also identified as the primary driver of DALYs. Regarding LC, smoking and high alcohol use were the leading risk factors, with smoking contributing to over 60% of the DALYs in all five nations. Similarly, the burden of OPC was primarily driven by smoking and high alcohol use. Notably, China had the highest proportion of OPC DALYs attributable to smoking (52.00%), while the Republic of Korea exhibited the highest contribution from high alcohol use (33.50%) (Fig 6).

### Forecasting trends in HNC burden from 2023 to 2038

**Fig 7:**
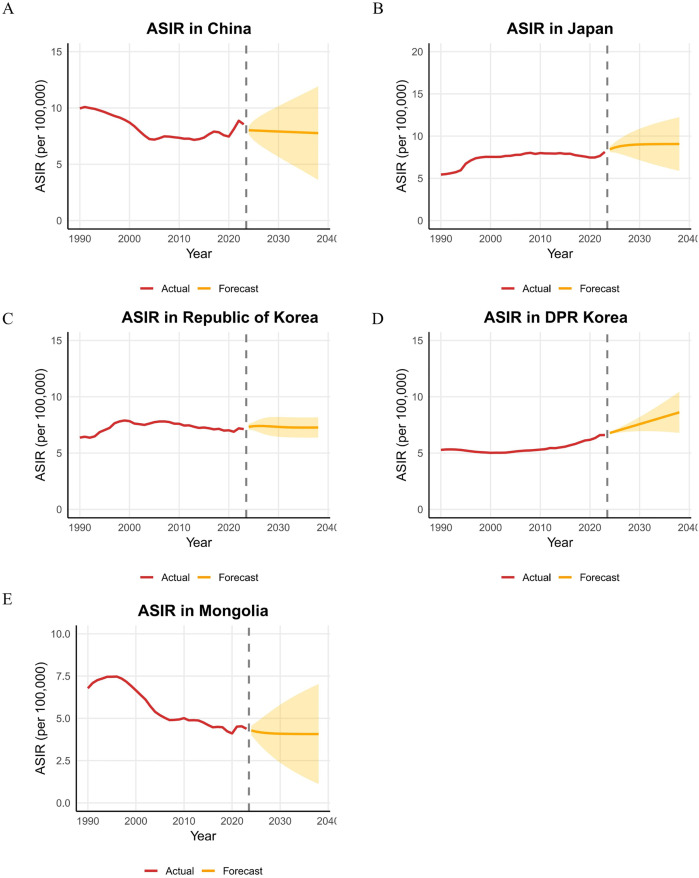
Temporal trends of ASIR of head and neck cancer in five East Asian countries from 1990 to 2023, with projections for 2024–2038. **A.** China, **B.** Japan, **C.** Republic of Korea, **D.** Democratic People’s Republic of Korea, **E.** Mongolia; ASIR, age-standardized incidence rate.

Projections from 2023 to 2038 indicate divergent trends in the age-standardized incidence and mortality rates of HNC across East Asia. For ASIR, a decline is anticipated in China (from 8.55 to 7.77 per 100,000, −9.12%) and Mongolia (from 4.38 to 4.07 per 100,000, −7.08%), whereas increases are projected for Japan (from 8.13 to 9.06 per 100,000, + 11.44%), the Republic of Korea (from 7.13 to 7.27 per 100,000, + 1.96%), and notably, the DPR of Korea (from 6.60 to 8.61 per 100,000, + 30.45%). Regarding ASMR, substantial reductions are forecasted for China (−50.58%), the Republic of Korea (−34.58%), Japan (−11.60%), and Mongolia (−11.68%). In contrast, the Democratic People’s Republic of Korea is projected to experience a 26.1% increase in ASMR. By 2038, East Asia is expected to exhibit significant heterogeneity in HNC burden: while China demonstrates a favorable pattern of concurrently declining incidence and mortality, Japan and the DPR of Korea face the challenge of rising incidence; moreover, the DPR of Korea is projected to have the highest mortality rate, contrasting with the steepest decline observed in China (Fig 7 and S7 Fig).

## Discussion

Based on the GBD 2023, this study systematically evaluated the HNC burden and its long-term trends in five East Asian countries from 1990 to 2023. The findings reveal significant heterogeneity in the burden across the region. Although the absolute number of cases increased universally due to population growth and aging, trends in age-standardized rates diverged markedly. China demonstrated substantial declines in ASIR, ASMR, and ASDALYR, indicating effective disease control. In contrast, Japan exhibited concerning increases in both ASIR and ASMR. The Republic of Korea maintained a stable ASIR while achieving a significant reduction in ASMR. Mongolia showed considerable declines across all burden indicators, whereas the DPR of Korea displayed slight upward trends in both incidence and mortality.

Age- and sex-stratified analyses revealed two dominant patterns: a consistently higher burden in males and a pronounced aging of the disease burden. The male predominance in HNC incidence and mortality across all countries is fundamentally rooted in historically gender-specific disparities in risk factor exposure. In East Asian societies, the significantly higher prevalence of smoking and alcohol consumption among males has been the primary behavioral driver of their elevated risk [29,30]. Region-specific habits, such as the higher prevalence of betel quid chewing among males in parts of China, have further intensified this gender gap [31]. In addition, men are exposed to occupational carcinogens more frequently than women, significantly contributing to the development of HNC [32]. Concurrently, a clear aging trend was observed, particularly in China, Japan, and the Republic of Korea, where the peak age for both age-specific incidence and mortality has shifted markedly toward older populations. This progression is closely synchronized with rapid demographic aging in these nations. Aging not only extends the duration of risk exposure but also reflects a cohort effect, where populations heavily exposed to potent risk factors like tobacco in their earlier years are now entering the high-risk age brackets for cancer development [33,34]. This effect is most evident in Japan, where the peak age-specific incidence has shifted to the 95 + age group, posing unique challenges for managing cancer in the very elderly. In contrast, the burden in the DPR of Korea and Mongolia remains concentrated in younger and middle-aged adults, a pattern more typical of regions in earlier stages of epidemiological transition, potentially linked to earlier risk exposure and a shorter life expectancy [35].

The significant decline in the age-standardized burden of HNC in China results from the synergistic effects of public health interventions and advancements in diagnostic and therapeutic technologies. At the level of risk factor control, national initiatives such as the “Healthy China 2030” strategy, alongside sustained anti-smoking campaigns and public education on the harms of excessive alcohol consumption [36–38], have likely contributed to a mitigation of population-level risk exposure. Concurrently, progress in early diagnosis and treatment has played an important role, including the adoption of plasma Epstein-Barr virus (EBV) DNA testing for NPC screening in high-risk populations [39], thereby facilitating earlier diagnosis. For patients with locoregionally advanced disease, optimized multimodal treatment strategies integrating surgery, radiotherapy, and chemotherapy have become standard. In the management of recurrent or metastatic HNC, targeted therapies, exemplified by the anti-EGFR monoclonal antibody cetuximab in combination with radiotherapy or platinum-based chemotherapy, have demonstrated survival benefits [40]. Recent clinical studies on immune checkpoint inhibitors, including pembrolizumab and nivolumab, have reported favorable results in the management of HNC [41]. However, despite the favorable trend in standardized rates at the macro level, the continued increase in the absolute burden of LOC reveals an unresolved challenge. This points to persistent, region-specific risks such as betel quid chewing in provinces like Hunan and Hainan [31,42], alongside the ongoing emergence of new cases from aging male cohorts with a history of heavy smoking and alcohol use. Furthermore, studies suggest that long-term adverse dietary habits prevalent in China, including high intake of preserved foods leading to the accumulation of potent carcinogenic nitrosamines, may directly damage oral mucosal DNA and elevate mutational risk over time, thereby contributing to the burden of LOC [43].

The marked increase in HNC burden, particularly for oropharyngeal cancer, in Japan and the Republic of Korea reflects the growing role of HPV infection alongside established tobacco-related risks. Of particular concern, Japan has experienced a sustained increase in both age‑standardized incidence and mortality rates over the most recent years. This continued upward trend may be attributable to multiple converging factors, including a persistently high prevalence of HPV‑related oropharyngeal cancers, delayed uptake of HPV vaccination especially among males, and the aging population, which together amplify the overall burden [44,45]. However, a study [46] showed that the ASMR of HNC in Japan decreased from 1999 to 2019. This divergence might be due to differences in data sources and time periods. This study includes the latest trends (from 2020 to 2023), as well as differences in the GBD data modeling methods and age standardization methods. The substantial increase in OPC-related DALYs in both countries suggests a growing impact of HPV infection in these developed East Asian economies, likely driven by evolving sexual behaviors and circulating oncogenic HPV genotypes [47]. Expanding HPV vaccination to both adolescent males and females represents a key preventive strategy; although both countries have implemented female vaccination [48], improving male coverage remains a public health priority, explicitly integrating it into national cancer control plans for oropharyngeal cancer prevention and improving male vaccination rates remains a critical public health priority. In contrast, resource-limited settings like Mongolia and the DPR of Korea face distinct gaps in screening capacity and treatment access [49].

The association between HNC burden and SDI reveals that higher development levels associate with improved survival, while China’s burden exceeding SDI-predicted levels likely reflects cumulative historical tobacco exposure. Risk attribution confirms smoking as the leading factor for HNC across East Asia, suggesting that tobacco control remains important to burden reduction efforts. Tobacco smoke contains approximately 70 established carcinogens that promote carcinogenesis through multiple interrelated mechanisms, including the formation of DNA-damaging adducts, chronic inflammation, and immunosuppression [50]. Decomposition and forecast analyses project continued regional divergence, with aging populations sustaining tobacco-attributable burden.

The substantial heterogeneity in HNC burden across East Asia underscores the urgent need for a targeted and multifaceted strategy. At the primary prevention level, intensifying tobacco and alcohol control remains paramount, particularly in high-burden countries like China. This must be complemented by targeted legislation and public education against betel quid chewing in endemic regions. Concurrently, in developed nations such as Japan and the Republic of Korea, prioritizing gender-neutral HPV vaccination programs is essential to curb the rising incidence of HPV-associated oropharyngeal cancer. Enhancing early detection requires a dual approach: raising awareness of early symptoms among the public and primary care practitioners, and implementing cost-effective screening for high-risk groups, such as long-term smokers and drinkers [51]. In Southern China, integrating plasma EBV DNA testing into regional screening programs for NPC shows significant promise [52]. Furthermore, the application of artificial intelligence for the analysis of medical images and health records offers a scalable strategy to improve early diagnosis, especially in resource-limited settings [53]. Optimizing treatment outcomes requires the promotion of standardized, multidisciplinary care models to ensure access to optimal therapy combinations, including surgery, radiotherapy, and systemic treatments. A critical challenge in many parts of Asia, particularly in low- and middle-income areas, remains limited access to radiotherapy [54]; addressing this infrastructure gap is essential for improving survival. Finally, fostering regional collaboration is imperative. Strengthening data sharing and cooperative research on HPV epidemiology, biomarker discovery, and novel therapies will accelerate progress. International support through technical assistance and capacity building is also crucial to enhance foundational cancer care capabilities in less-resourced settings, ensuring a more equitable regional response to the HNC challenge.

Our findings should be interpreted considering several inherent limitations. First, data quality disparities across countries, particularly in resource-limited settings like Mongolia and the DPR of Korea, may affect the precision of burden estimates. Second, the analysis only encompasses four major HNC subtypes, excluding rarer anatomical sites. Third, the descriptive design of this study mainly presents trend associations at the group level, fails to establish causal connections at the individual level, and cannot fully control the influence of confounding factors. Fourth, GBD 2023 does not include HPV infection as a risk factor, which precludes a formal assessment of its contribution to the observed trends in oropharyngeal cancer. Finally, the ARIMA projections assume historical patterns will persist and may not account for future healthcare innovations or policy shifts. The results offer valuable insights for health policy but should be interpreted with an understanding of these inherent methodological limitations and data uncertainties.

## Conclusion

This study delineates the substantial heterogeneity and evolving patterns of HNC burden across five East Asian countries from 1990 to 2023. The diverging national trajectories are shaped by the complex interplay of traditional risk factors, the emerging impact of HPV infection, and profound demographic shifts. These findings highlight the critical need for differentiated, evidence-based national strategies to achieve effective prevention, early detection, and equitable treatment. Addressing the dual challenge of legacy risks and new epidemiological threats is essential for reducing the future burden of HNC in the region.

## Supporting information

S1 Fig Comparison of DALYs attributed to head and neck cancer subtypes across five East Asian countries in 2023 based on global burden of disease. DALYs, disability-adjusted life years. DPR of Korea, the Democratic People’s Republic of Korea.
(TIF)

S2 Fig Number of head and neck cancer mortality across different age groups in five East Asian countries in 1990 and 2023. (TIF)

S3 Fig Comparison of ASMR for different age groups in five East Asian countries in 1990 and 2023. ASMR, age-standardized mortality rate.
(TIF)

S4 Fig Joinpoint regression analysis of ASMR of head and neck cancer in five East Asian countries from 1990 to 2023. (A) China, (B) Japan, (C) Republic of Korea, (D) Democratic People’s Republic of Korea, (E) Mongolia.*indicates a *P*-value less than 0.05. ASMR, age-standardized mortality rate.
(TIF)

S5 Fig The associations between the SDI and ASRs of head and neck cancer. ASRs, age-standardized rates; DPR of Korea, Democratic People’s Republic of Korea; SDI, Socio-Demographic Index.
(TIF)

S6 Fig Decomposition analysis of DALYs change of head and neck cancer from 1990 to 2023. DALYs, disability-adjusted life years.
(TIF)

S7 Fig Temporal trends of ASMR of head and neck cancer in five East Asian countries from 1990 to 2023, with projections for 2024–2038. A) China, B) Japan, C) Republic of Korea, D) Democratic People’s Republic of Korea, E) Mongolia. ASMR, age-standardized mortality rate.
(TIF)

S1 Table List of International Classification of Diseases (ICD) codes mapped to the Global Burden of Disease cause list for head and neck cancers. (DOCX)

S2 Table DALYs of different subtypes of head and neck cancers in five East Asian countries in 1990 and 2023. (DOCX)

S3 Table Joinpoint regression analysis of head and neck cancer in five East Asian countries from 1990 to 2023. (DOCX)

S4 Table Results of Decomposition Analysis for HNC DALYs. (DOCX)

## References

[1] Chow LQM. Head and Neck Cancer. N Engl J Med. 2020;382(1):60–72. doi: 10.1056/NEJMra1715715 31893516

[2] Bray F, Laversanne M, Sung H, Ferlay J, Siegel RL, Soerjomataram I. Global cancer statistics 2022: GLOBOCAN estimates of incidence and mortality worldwide for 36 cancers in 185 countries. CA Cancer J Clin. 2024;74(3):229–63. doi: 10.3322/caac.21834 38572751

[3] Hashibe M, Brennan P, Chuang S-C, Boccia S, Castellsague X, Chen C, et al. Interaction between tobacco and alcohol use and the risk of head and neck cancer: pooled analysis in the International Head and Neck Cancer Epidemiology Consortium. Cancer Epidemiol Biomarkers Prev. 2009;18(2):541–50. doi: 10.1158/1055-9965.EPI-08-0347 19190158 PMC3051410

[4] Gormley M, Creaney G, Schache A, Ingarfield K, Conway DI. Reviewing the epidemiology of head and neck cancer: definitions, trends and risk factors. Br Dent J. 2022;233(9):780–6. doi: 10.1038/s41415-022-5166-x 36369568 PMC9652141

[5] Mehanna H, Beech T, Nicholson T, El-Hariry I, McConkey C, Paleri V, et al. Prevalence of human papillomavirus in oropharyngeal and nonoropharyngeal head and neck cancer--systematic review and meta-analysis of trends by time and region. Head Neck. 2013;35(5):747–55. doi: 10.1002/hed.22015 22267298

[6] Budach V, Tinhofer I. Novel prognostic clinical factors and biomarkers for outcome prediction in head and neck cancer: a systematic review. Lancet Oncol. 2019;20(6):e313–26. doi: 10.1016/S1470-2045(19)30177-9 31162105

[7] Schindler A, Denaro N, Russi EG, Pizzorni N, Bossi P, Merlotti A, et al. Dysphagia in head and neck cancer patients treated with radiotherapy and systemic therapies: Literature review and consensus. Crit Rev Oncol Hematol. 2015;96(2):372–84. doi: 10.1016/j.critrevonc.2015.06.005 26141260

[8] Bossi P, Giusti R, Tarsitano A, Airoldi M, De Sanctis V, Caspiani O, et al. The point of pain in head and neck cancer. Crit Rev Oncol Hematol. 2019;138:51–9. doi: 10.1016/j.critrevonc.2019.04.001 31092385

[9] Luryi AL, Yarbrough WG, Niccolai LM, Roser S, Reed SG, Nathan C-AO, et al. Public awareness of head and neck cancers: a cross-sectional survey. JAMA Otolaryngol Head Neck Surg. 2014;140(7):639–46. doi: 10.1001/jamaoto.2014.867 24902640

[10] Lin J, Xie B, Yi X, Wu S, Ji Y, Xu E, et al. Global burden and trends of common head and neck cancers between 1990 and 2021: findings from the Global Burden of Disease Study 2021. BMC Oral Health. 2025;25(1):1773. doi: 10.1186/s12903-025-07140-6 41225462 PMC12613622

[11] Ding M, Yu S, Chen Y, Liu Y, Xuan F. Space-time analysis of head and neck cancer in Asia and its 34 countries and territories (1990-2021): Implications from the Global Burden of Disease Study 2021. PLoS One. 2025;20(6):e0326177. doi: 10.1371/journal.pone.0326177 40526708 PMC12173354

[12] Zhang S, Sun K, Zheng R, Zeng H, Wang S, Chen R, et al. Cancer incidence and mortality in China, 2015. J Natl Cancer Cent. 2021;1(1):2–11. doi: 10.1016/j.jncc.2020.12.001 39036787 PMC11256613

[13] Menezes FDS, Fernandes GA, Antunes JLF, Villa LL, Toporcov TN. Global incidence trends in head and neck cancer for HPV-related and -unrelated subsites: A systematic review of population-based studies. Oral Oncol. 2021;115:105177. doi: 10.1016/j.oraloncology.2020.105177 33561611

[14] Burden of 375 diseases and injuries, risk-attributable burden of 88 risk factors, and healthy life expectancy in 204 countries and territories, including 660 subnational locations, 1990-2023: a systematic analysis for the Global Burden of Disease Study 2023. Lancet. 2025;406(10513):1873–922. doi: 10.1016/s0140-6736(25)01637-x 41092926 PMC12535840

[15] Global age-sex-specific all-cause mortality and life expectancy estimates for 204 countries and territories and 660 subnational locations, 1950-2023: a demographic analysis for the Global Burden of Disease Study 2023. Lancet. 2025;406(10513):1731–810. doi: 10.1016/s0140-6736(25)01330-3 41092927 PMC12535839

[16] Cagney J, Spencer C, Flor L, Herbert M, Khalil M, O’Connell E, et al. Prevalence of sexual violence against children and age at first exposure: a global analysis by location, age, and sex (1990-2023). Lancet. 2025;405(10492):1817–36. doi: 10.1016/s0140-6736(25)00311-3 40347967 PMC12100463

[17] Global, regional, and national prevalence of kidney failure with replacement therapy and associated aetiologies, 1990-2023: a systematic analysis for the Global Burden of Disease Study 2023. Lancet Glob Health. 2025;13(8):e1378–95. doi: 10.1016/s2214-109x(25)00198-6 40712611

[18] GBD 2023 Cancer Collaborators. The global, regional, and national burden of cancer, 1990-2023, with forecasts to 2050: a systematic analysis for the Global Burden of Disease Study 2023. Lancet. 2025;406(10512):1565–86. doi: 10.1016/S0140-6736(25)01635-6 41015051 PMC12687902

[19] Yang X, Zhang T, Zhang X, Chu C, Sang S. Global burden of lung cancer attributable to ambient fine particulate matter pollution in 204 countries and territories, 1990-2019. Environ Res. 2022;204(Pt A):112023. doi: 10.1016/j.envres.2021.112023 34520750

[20] Su Z, Zou Z, Hay SI, Liu Y, Li S, Chen H, et al. Global, regional, and national time trends in mortality for congenital heart disease, 1990-2019: An age-period-cohort analysis for the Global Burden of Disease 2019 study. EClinicalMedicine. 2022;43:101249. doi: 10.1016/j.eclinm.2021.101249 35059612 PMC8760503

[21] Bell A. Age period cohort analysis: a review of what we should and shouldn’t do. Ann Hum Biol. 2020;47(2):208–17. doi: 10.1080/03014460.2019.1707872 32429768

[22] Holford TR. Approaches to fitting age-period-cohort models with unequal intervals. Stat Med. 2006;25(6):977–93. doi: 10.1002/sim.2253 16143994

[23] Rosenberg PS, Check DP, Anderson WF. A web tool for age-period-cohort analysis of cancer incidence and mortality rates. Cancer Epidemiol Biomarkers Prev. 2014;23(11):2296–302. doi: 10.1158/1055-9965.EPI-14-0300 25146089 PMC4221491

[24] Liang X, Lyu Y, Li J, Li Y, Chi C. Global, regional, and national burden of preterm birth, 1990-2021: a systematic analysis from the global burden of disease study 2021. EClinicalMedicine. 2024;76:102840. doi: 10.1016/j.eclinm.2024.102840 39386159 PMC11462015

[25] Das Gupta P. Standardization and decomposition of rates from cross-classified data. Genus. 1994;50(3–4):171–96. 12319256

[26] Zhang T, Chen H, Yin X, He Q, Man J, Yang X, et al. Changing trends of disease burden of gastric cancer in China from 1990 to 2019 and its predictions: Findings from Global Burden of Disease Study. Chin J Cancer Res. 2021;33(1):11–26. doi: 10.21147/j.issn.1000-9604.2021.01.02 33707924 PMC7941685

[27] Yang P, Huang W, Xu Y, Teng Y, Shu P. Trends and projections of the burden of gastric cancer in China and G20 countries: a comparative study based on the global burden of disease database 2021. Int J Surg. 2025;111(7):4854–65. doi: 10.1097/JS9.0000000000002464 40359560

[28] Lin S, Liu R, Zhong G, Lyu P, Liu L, Zhang B, et al. Burden and trends of diabetic kidney disease in East Asia, 1990-2038: An analysis of the global burden of disease study 2023. Diabetes Metab Syndr. 2025;19(11):103333. doi: 10.1016/j.dsx.2025.103333 41317674

[29] Wen X, Liao Y, Li J, Zhu Q, Lin X, Han B, et al. Over one-third of cancer cases and two-fifths of cancer deaths in southern China are preventable: Insights from the latest representative population-based cancer registry data and risk factor survey. Int J Cancer. 2026;158(4):909–23. doi: 10.1002/ijc.70114 40900439 PMC12712366

[30] Lee Y-CA, Li S, Chen Y, Li Q, Chen C-J, Hsu W-L, et al. Tobacco smoking, alcohol drinking, betel quid chewing, and the risk of head and neck cancer in an East Asian population. Head Neck. 2019;41(1):92–102. doi: 10.1002/hed.25383 30552826

[31] Zhang X, Reichart PA. A review of betel quid chewing, oral cancer and precancer in Mainland China. Oral Oncol. 2007;43(5):424–30. doi: 10.1016/j.oraloncology.2006.08.010 17258497

[32] Sassano M, Seyyedsalehi MS, Siea AC, Boffetta P. Occupational arsenic exposure and digestive and head and neck cancers: A systematic review and meta-analysis. Environ Res. 2024;260:119643. doi: 10.1016/j.envres.2024.119643 39053758

[33] Gorasso V, Vandevijvere S, Van der Heyden J, Pelgrims I, Hilderink H, Nusselder W, et al. The incremental healthcare cost associated with cancer in Belgium: A registry-based data analysis. Cancer Med. 2024;13(3):e6659. doi: 10.1002/cam4.6659 38268318 PMC10905540

[34] Xie L, Qian Y, Liu Y, Li Y, Jia S, Yu H, et al. Distinctive lung cancer incidence trends among men and women attributable to the period effect in Shanghai: An analysis spanning 42 years. Cancer Med. 2020;9(8):2930–9. 10.1002/cam4.2917 32073760 PMC7163103

[35] Guo T, Zhu W, Hui Y, Wang Y, Zhou T, Shen W. The burden of pancreatic cancer in five East Asian countries from 1990 to 2021 and its prediction up to 2036: A systemic analysis of the Global Burden of Diseases Study 2021. Cancer Med. 2025;14(23):e70656. doi: 10.1002/cam4.70656 41355376 PMC12683073

[36] Sun D, Pang Y, Lyu J, Li L. Current Progress and Challenges to Tobacco Control in China. China CDC Wkly. 2022;4(6):101–5. doi: 10.46234/ccdcw2022.020 35186379 PMC8844520

[37] Li X, Galea G. Healthy China 2030: an opportunity for tobacco control. Lancet. 2019;394(10204):1123–5. doi: 10.1016/s0140-6736(19)32048-3 31571589

[38] Chen P, Li F, Harmer P. Healthy China 2030: moving from blueprint to action with a new focus on public health. Lancet Public Health. 2019;4(9):e447. doi: 10.1016/S2468-2667(19)30160-4 31493840

[39] Tsao SW, Tsang CM, Lo KW. Epstein-Barr virus infection and nasopharyngeal carcinoma. Philos Trans R Soc Lond B Biol Sci. 2017;372(1732):20160270. doi: 10.1098/rstb.2016.0270 28893937 PMC5597737

[40] Forman R, Deshpande H, Burtness B, Bhatia AK. Efficacy and toxicity of weekly paclitaxel, carboplatin, and cetuximab as induction chemotherapy or in cases of metastases or relapse for head and neck cancer with a focus on elderly or frail patients. Head Neck. 2022;44(8):1777–86. doi: 10.1002/hed.27077 35488876

[41] Vallianou NG, Evangelopoulos A, Kounatidis D, Panagopoulos F, Geladari E, Karampela I. Current Oncology Reports. 2023;25(8):897–912. doi: 10.1007/s11912-023-01425-1 37213060

[42] Sreekissoon S, Wang D, Wang Y, Wang C, Neda TS, Zhu M, et al. Distinctive tumor biology of oral squamous cell carcinoma associated with oral submucous fibrosis and its suppressive immune microenvironment. BMC Oral Health. 2025;26(1):347. doi: 10.1186/s12903-025-07464-3 41366766 PMC12922367

[43] Bulanda S, Lau K, Nowak A, Łyko-Morawska D, Kotylak A, Janoszka B. The risk of oral cancer and the high consumption of thermally processed meat containing mutagenic and carcinogenic compounds. Nutrients. 2024;16(7). doi: 10.3390/nu16071084 38613117 PMC11013896

[44] Kawakita D, Oze I, Iwasaki S, Matsuda T, Matsuo K, Ito H. Trends in the incidence of head and neck cancer by subsite between 1993 and 2015 in Japan. Cancer Med. 2022;11(6):1553–60. doi: 10.1002/cam4.4539 35029329 PMC8921930

[45] Nibu K-I, Oridate N, Saito Y, Roset M, Forés Maresma M, Cuadras D, et al. Human papillomavirus-driven head and neck cancers in Japan during 2008-2009 and 2018-2019: The BROADEN study. Cancer Sci. 2024;115(8):2808–18. doi: 10.1111/cas.16230 38847353 PMC11309946

[46] Higashionna T, Harada K, Maruo A, Niimura T, Tan E, Vu QT, et al. Trends in Head and Neck Cancer Mortality from 1999 to 2019 in Japan: An Observational Analysis. Cancers (Basel). 2023;15(15):3786. doi: 10.3390/cancers15153786 37568602 PMC10417308

[47] Taberna M, Mena M, Pavón MA, Alemany L, Gillison ML, Mesía R. Human papillomavirus-related oropharyngeal cancer. Ann Oncol. 2017;28(10):2386–98. doi: 10.1093/annonc/mdx304 28633362

[48] Miyagi E. Human papillomavirus (HPV) vaccination in Japan. J Obstet Gynaecol Res. 2024;50 Suppl 1:65–71. doi: 10.1111/jog.16020 38979785

[49] Jigjidsuren A, Byambaa T, Altangerel E, Batbaatar S, Saw YM, Kariya T, et al. Free and universal access to primary healthcare in Mongolia: the service availability and readiness assessment. BMC Health Serv Res. 2019;19(1):129. doi: 10.1186/s12913-019-3932-5 30786897 PMC6381625

[50] Jun S, Park H, Kim U-J, Lee HA, Park B, Lee SY, et al. The Combined Effects of Alcohol Consumption and Smoking on Cancer Risk by Exposure Level: A Systematic Review and Meta-Analysis. J Korean Med Sci. 2024;39(22):e185. doi: 10.3346/jkms.2024.39.e185 38859742 PMC11164648

[51] Nagao T, Warnakulasuriya S. Screening for oral cancer: Future prospects, research and policy development for Asia. Oral Oncol. 2020;105:104632. doi: 10.1016/j.oraloncology.2020.104632 32315954

[52] Sykes EA, Weisbrod N, Rival E, Haque A, Fu R, Eskander A. Methods, Detection Rates, and Survival Outcomes of Screening for Head and Neck Cancers. JAMA Otolaryngol Head Neck Surg. 2023;149(11):1047. doi: 10.1001/jamaoto.2023.3010 37796524

[53] Mahmood H, Shaban M, Rajpoot N, Khurram SA. Artificial Intelligence-based methods in head and neck cancer diagnosis: an overview. Br J Cancer. 2021;124(12):1934–40. doi: 10.1038/s41416-021-01386-x 33875821 PMC8184820

[54] Abu Awwad D, Shafiq J, Delaney GP, Anacak Y, Bray F, Flores JA, et al. Current and projected gaps in the availability of radiotherapy in the Asia-Pacific region: a country income-group analysis. Lancet Oncol. 2024;25(2):225–34. doi: 10.1016/S1470-2045(23)00619-8 38301690

