# Supplementary figures and images for: Burden of head and neck cancers in five East Asian countries from 1990 to 2023: Observation, comparison, and forecast from the global burden of disease study 2023
Source: PLoS One. 2026 May 15;21(5):e0349297. doi: 10.1371/journal.pone.0349297 (PMC13178879; doi:10.1371/journal.pone.0349297)

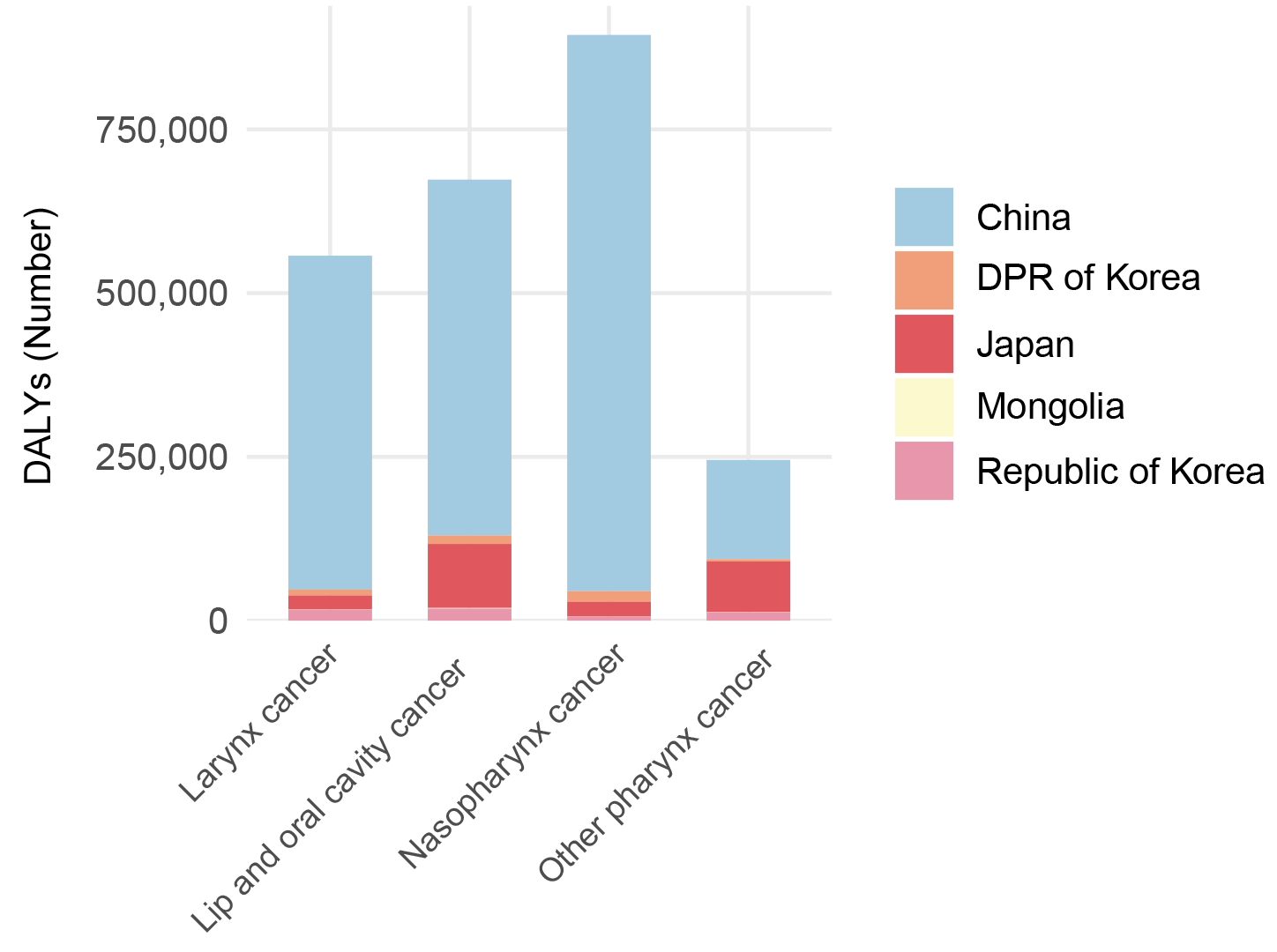

Supplement: S1 Fig — DALYs, disability-adjusted life years. DPR of Korea, the Democratic People’s Republic of Korea. (TIF) [file pone.0349297.s001.tif]

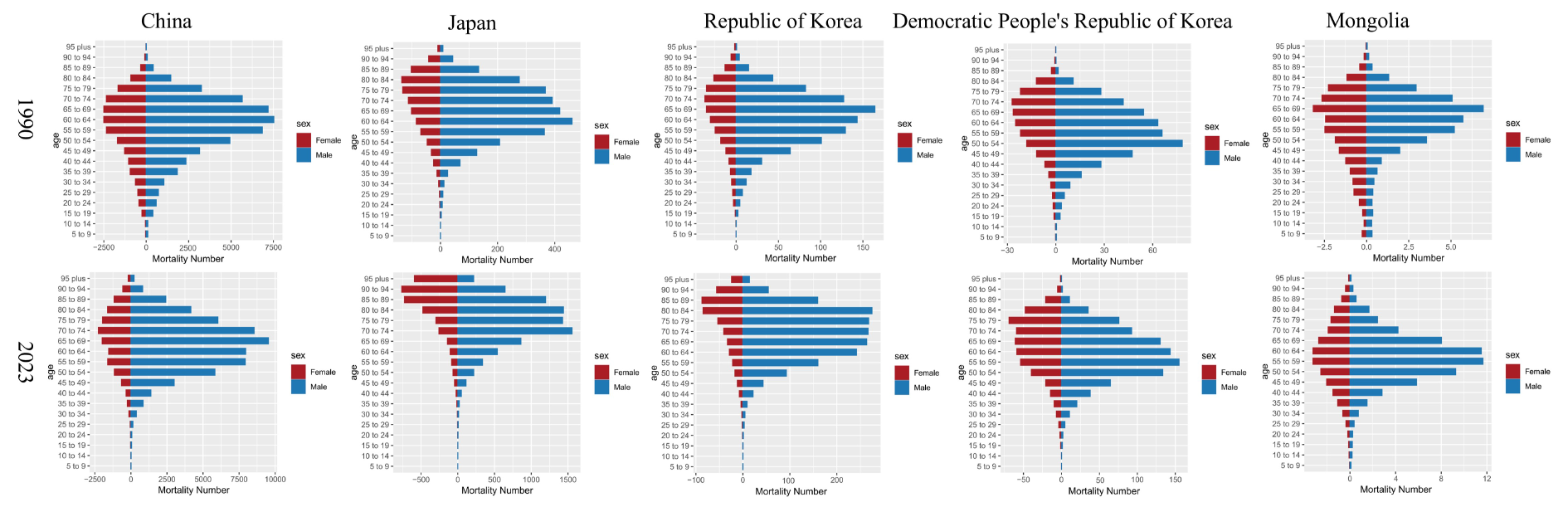

Supplement: S2 Fig — (TIF) [file pone.0349297.s002.tif]

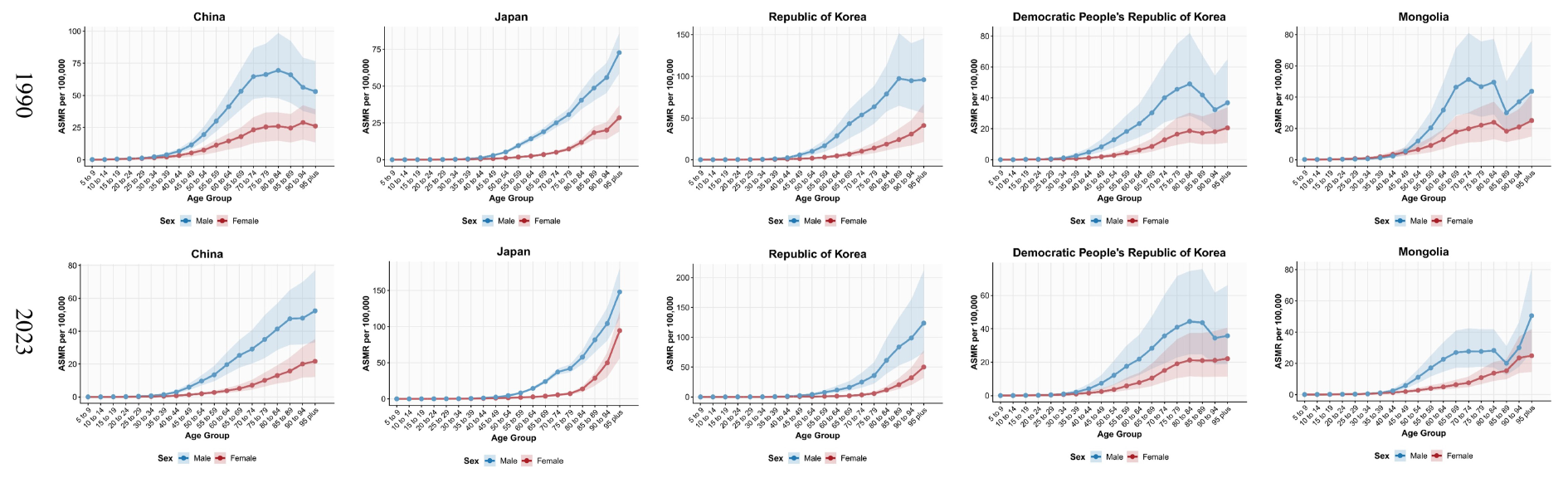

Supplement: S3 Fig — ASMR, age-standardized mortality rate. (TIF) [file pone.0349297.s003.tif]

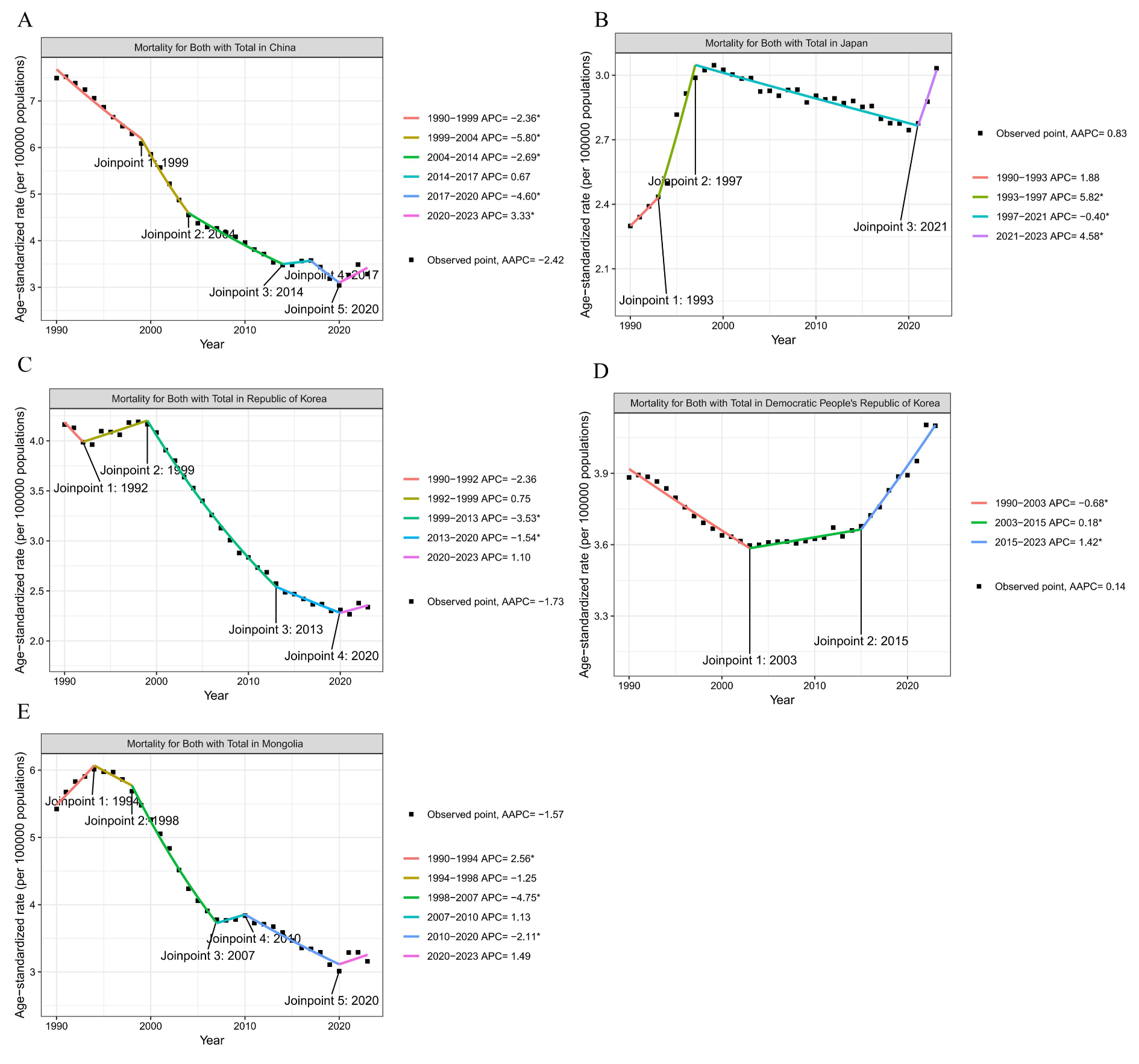

Supplement: S4 Fig — (A) China, (B) Japan, (C) Republic of Korea, (D) Democratic People’s Republic of Korea, (E) Mongolia.*indicates a P-value less than 0.05. ASMR, age-standardized mortality rate. (TIF) [file pone.0349297.s004.tif]

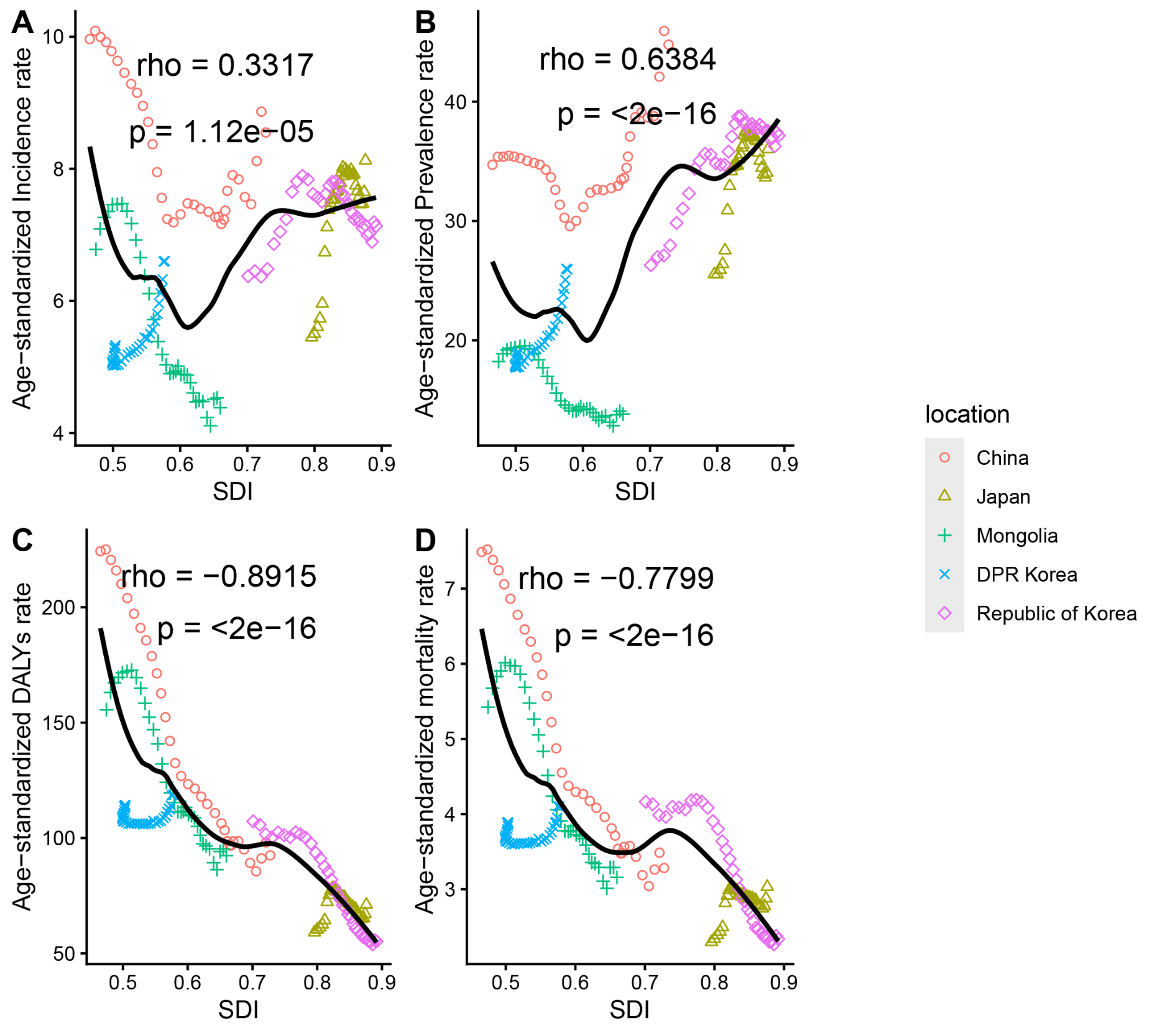

Supplement: S5 Fig — ASRs, age-standardized rates; DPR of Korea, Democratic People’s Republic of Korea; SDI, Socio-Demographic Index. (TIF) [file pone.0349297.s005.tif]

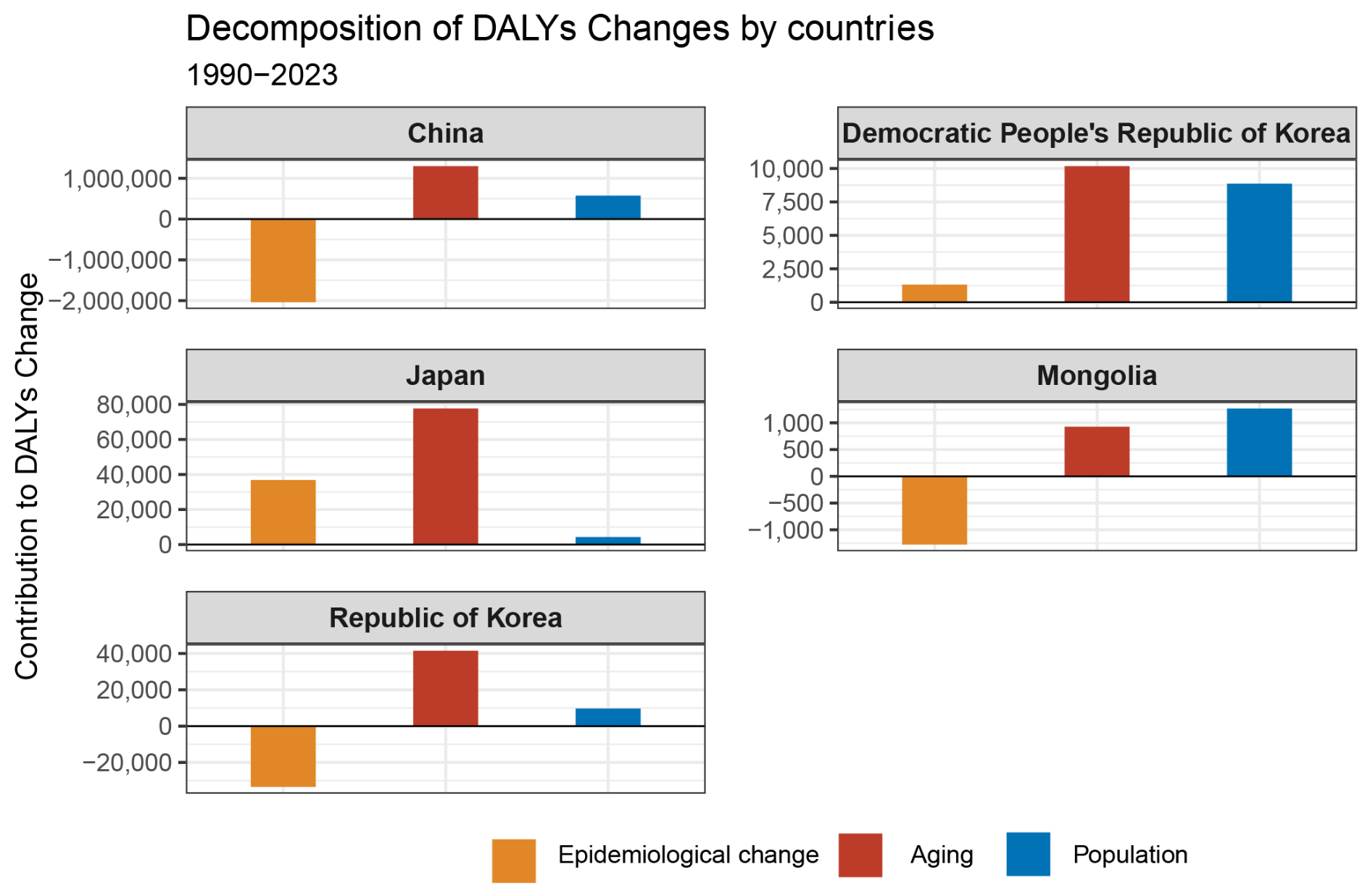

Supplement: S6 Fig — DALYs, disability-adjusted life years. (TIF) [file pone.0349297.s006.tif]

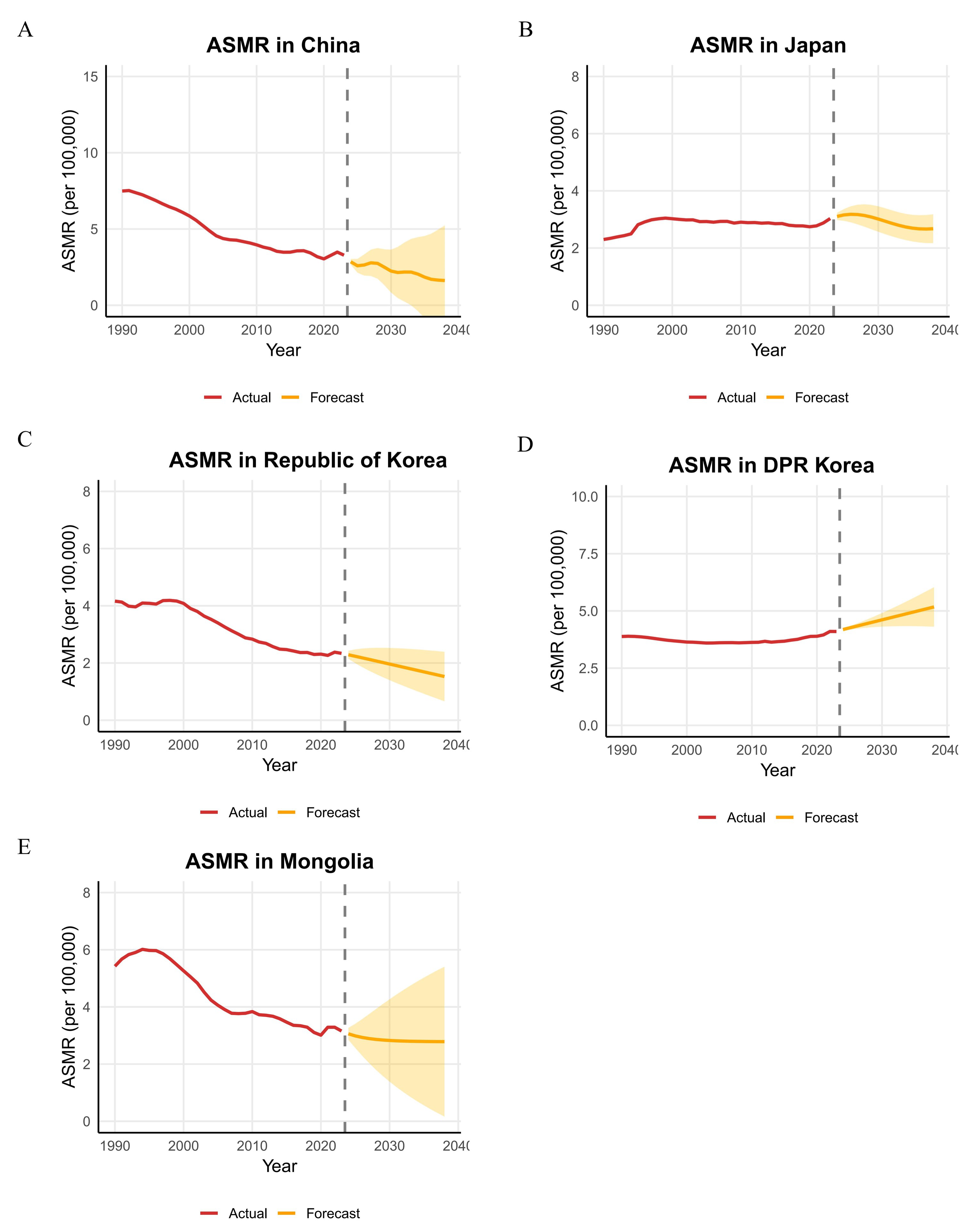

Supplement: S7 Fig — A) China, B) Japan, C) Republic of Korea, D) Democratic People’s Republic of Korea, E) Mongolia. ASMR, age-standardized mortality rate. (TIF) [file pone.0349297.s007.tif]
