# Supplementary material for: Burden of head and neck cancers in five East Asian countries from 1990 to 2023: Observation, comparison, and forecast from the global burden of disease study 2023
Source: PLoS One. 2026 May 15;21(5):e0349297. doi: 10.1371/journal.pone.0349297 (PMC13178879; doi:10.1371/journal.pone.0349297)
Supplement: S1 Table — (DOCX) [file pone.0349297.s008.docx]

**Supplementary Table S1** List of International Classification of Diseases (ICD) codes mapped to the Global Burden of Disease cause list for head and neck cancers

| **Cause** | **ICD10** | **ICD9** |
| --- | --- | --- |
| Nasopharynx cancer | C11-C11.9, D10.6 | 147-147.9, 210.7-210.9 |
| Larynx cancer | C32-C32.9, D02.0, D14.1, D38.0 | 161-161.9, 212.1, 231.0, 235.6 |
| Lip and oral cavity cancer | C00-C08.9, D10.0-D10.5, D11-D11.9 | 140-145.9, 210.0-210.6, 235.0 |
| Other pharynx cancer | C09-C10.9, C12-C13.9, D10.7 | 146-146.9, 148-148.9 |
