# Supplementary material for: Burden of head and neck cancers in five East Asian countries from 1990 to 2023: Observation, comparison, and forecast from the global burden of disease study 2023
Source: PLoS One. 2026 May 15;21(5):e0349297. doi: 10.1371/journal.pone.0349297 (PMC13178879; doi:10.1371/journal.pone.0349297)
Supplement: S2 Table — (DOCX) [file pone.0349297.s009.docx]

**Supplementary Table S2** DALYs of different subtypes of head and neck cancers in five East Asian countries in 1990 and 2023

| Subtype | Lip and oral cavity cancer | | | |
| --- | --- | --- | --- | --- |
|  |  | DALYs in 1990 | DALYs in 2023 | Change (%) |
| Country | Mongolia | 923 | 1,132 | +22.64 % |
|  | Republic of Korea | 9,508 | 18,443 | +94.00 % |
|  | China | 347,066 | 543,250 | +56.52 % |
|  | Democratic People’s Republic of Korea | 5,449 | 12,955 | +137.80 % |
|  | Japan | 42,677 | 97,524 | +128.50 % |
|  | Nasopharynx cancer | | | |
| Country | Mongolia | 342 | 483 | +41.23 % |
|  | Republic of Korea | 5,597 | 5,906 | +5.52 % |
|  | China | 1,370,696 | 849,171 | –38.04 % |
|  | Democratic People’s Republic of Korea | 10,102 | 16,061 | +59.00 % |
|  | Japan | 12,961 | 22,509 | +73.66 % |
|  | Larynx cancer | | | |
| Country | Mongolia | 187 | 499 | +166.84 % |
|  | Republic of Korea | 9 317 | 9 684 | +3.94 % |
|  | China | 262 459 | 398 126 | +51.69 % |
|  | Democratic People’s Republic of Korea | 2 770 | 5 889 | +112.60 % |
|  | Japan | 19 923 | 17 242 | –13.46 % |
|  | Other pharynx cancer | | | |
| Country | Mongolia | 282 | 517 | +83.33 % |
|  | Republic of Korea | 3 661 | 12 781 | +249.13 % |
|  | China | 132 850 | 151 579 | +14.10 % |
|  | Democratic People’s Republic of Korea | 1 679 | 3 534 | +110.48 % |
|  | Japan | 20 280 | 77 006 | +279.68 % |

DALYs, disability-adjusted life years.
