# Supplementary material for: Burden of head and neck cancers in five East Asian countries from 1990 to 2023: Observation, comparison, and forecast from the global burden of disease study 2023
Source: PLoS One. 2026 May 15;21(5):e0349297. doi: 10.1371/journal.pone.0349297 (PMC13178879; doi:10.1371/journal.pone.0349297)
Supplement: S3 Table — (DOCX) [file pone.0349297.s010.docx]

**Supplementary Table S3**. Joinpoint regression analysis of head and neck cancer in five East Asian countries from 1990 to 2023

| measure | location | Start year | End year | AAPC/APC | *P* |
| --- | --- | --- | --- | --- | --- |
| Incidence | China | 1990 | 2023 | -0.42 (-0.56 to -0.28) | *P*<0.05 |
|  | DPR of Korea | 1990 | 2023 | 0.7 (0.65 to 0.75) | *P*<0.05 |
|  | Japan | 1990 | 2023 | 1.21 (1.12 to 1.3) | *P*<0.05 |
|  | Mongolia | 1990 | 2023 | -1.31 (-1.46 to -1.18) | *P*<0.05 |
|  | Republic of Korea | 1990 | 2023 | 0.37 (0.29 to 0.44) | *P*<0.05 |
| Mortality | China | 1990 | 2023 | -2.42 (-2.54 to -2.3) | *P*<0.05 |
|  | DPR of Korea | 1990 | 2023 | 0.14 (0.11 to 0.17) | *P*<0.05 |
|  | Japan | 1990 | 2023 | 0.83 (0.73 to 0.93) | *P*<0.05 |
|  | Mongolia | 1990 | 2023 | -1.57 (-1.7 to -1.43) | *P*<0.05 |
|  | Republic of Korea | 1990 | 2023 | -1.73 (-1.81 to -1.61) | *P*<0.05 |
| Incidence | China | 1990 | 1999 | -1.25 (-1.71 to -0.55) | *P*<0.05 |
|  |  | 1999 | 2004 | -4.51 (-6.64 to -3.32) | *P*<0.05 |
|  |  | 2004 | 2020 | 0.36 (0.09 to 0.6) | *P*<0.05 |
|  |  | 2020 | 2023 | 5.02 (2.74 to 8.61) | *P*<0.05 |
|  | DPR of Korea | 1990 | 1992 | 0.49 (-0.65 to 1.25) | *P* = 0.41 |
|  |  | 1992 | 2001 | -0.77 (-1.11 to 0.81) | *P* = 0.14 |
|  |  | 2001 | 2015 | 0.77 (0.65 to 1.6) | *P*<0.05 |
|  |  | 2015 | 2023 | 2.31 (2.11 to 2.59) | *P*<0.05 |
|  | Japan | 1990 | 1993 | 1.75 (-0.09 to 3) | *P* = 0.055 |
|  |  | 1993 | 1997 | 6.87 (6.15 to 7.78) | *P*<0.05 |
|  |  | 1997 | 2013 | 0.48 (0.36 to 0.62) | *P*<0.05 |
|  |  | 2013 | 2021 | -1.01 (-1.58 to -0.71) | *P*<0.05 |
|  |  | 2021 | 2023 | 4.25 (2.12 to 5.68) | *P*<0.05 |
|  | Mongolia | 1990 | 1997 | 1.19 (0.27 to 2.06) | *P*<0.05 |
|  |  | 1997 | 2006 | -4.59 (-5.49 to -0.73) | *P*<0.05 |
|  |  | 2006 | 2012 | -0.22 (-4.59 to 2.27) | *P* = 0.70 |
|  |  | 2012 | 2020 | -1.85 (-4.22 to -1.17) | *P*<0.05 |
|  |  | 2020 | 2023 | 2.24 (-0.13 to 5.42) | *P* = 0.07 |
|  | Republic of Korea | 1990 | 1993 | 0.68 (-0.82 to 1.76) | *P* = 0.16 |
|  |  | 1993 | 1998 | 4 (3.46 to 5.02) | *P*<0.05 |
|  |  | 1998 | 2003 | -1.03 (-1.98 to -0.56) | *P*<0.05 |
|  |  | 2003 | 2006 | 1.36 (0.28 to 1.9) | *P*<0.05 |
|  |  | 2006 | 2021 | -0.81 (-1.05 to -0.73) | *P*<0.05 |
|  |  | 2021 | 2023 | 2.01 (-0.04 to 2.93) | *P* = 0.055 |
| Mortality | China | 1990 | 1999 | -2.36 (-2.82 to -1.78) | *P*<0.05 |
|  |  | 1999 | 2004 | -5.8 (-7.63 to -4.64) | *P*<0.05 |
|  |  | 2004 | 2014 | -2.69 (-3.43 to -2.24) | *P*<0.05 |
|  |  | 2014 | 2017 | 0.67 (-1.64 to 1.81) | *P* = 0.53 |
|  |  | 2017 | 2020 | -4.6 (-5.86 to -2.49) | *P*<0.05 |
|  |  | 2020 | 2023 | 3.33 (1.7 to 6.71) | *P*<0.05 |
|  | DPR of Korea | 1990 | 2003 | -0.68 (-0.77 to -0.6) | *P*<0.05 |
|  |  | 2003 | 2015 | 0.18 (0.07 to 0.29) | *P*<0.05 |
|  |  | 2015 | 2023 | 1.42 (1.26 to 1.62) | *P*<0.05 |
|  | Japan | 1990 | 1993 | 1.88 (-0.17 to 3.15) | *P* = 0.07 |
|  |  | 1993 | 1997 | 5.82 (4.91 to 6.83) | *P*<0.05 |
|  |  | 1997 | 2021 | -0.4 (-0.48 to -0.34) | *P*<0.05 |
|  |  | 2021 | 2023 | 4.58 (2.32 to 5.78) | *P*<0.05 |
|  | Mongolia | 1990 | 1994 | 2.56 (1.35 to 4.84) | *P*<0.05 |
|  |  | 1994 | 1998 | -1.25 (-4.14 to 0.25) | *P* = 0.09 |
|  |  | 1998 | 2007 | -4.75 (-5.75 to -4.25) | *P*<0.05 |
|  |  | 2007 | 2010 | 1.13 (-1.2 to 2.22) | *P* = 0.32 |
|  |  | 2010 | 2020 | -2.11 (-3.5 to -1.8) | *P*<0.05 |
|  |  | 2020 | 2023 | 1.49 (-0.54 to 4.3) | *P* = 0.13 |
|  | Republic of Korea | 1990 | 1992 | -2.36 (-3.49 to 0.1) | *P* = 0.06 |
|  |  | 1992 | 1999 | 0.75 (-1.99 to 1.79) | *P* = 0.06 |
|  |  | 1999 | 2013 | -3.53 (-3.79 to -3.31) | *P*<0.05 |
|  |  | 2013 | 2020 | -1.54 (-2.87 to -1) | *P*<0.05 |
|  |  | 2020 | 2023 | 1.1 (-0.36 to 2.93) | *P* = 0.12 |

AAPC, average annual percentage change; APC, annual percentage change; DPR of Korea, Democratic People's Republic of Korea
