# Supplementary material for: Burden of head and neck cancers in five East Asian countries from 1990 to 2023: Observation, comparison, and forecast from the global burden of disease study 2023
Source: PLoS One. 2026 May 15;21(5):e0349297. doi: 10.1371/journal.pone.0349297 (PMC13178879; doi:10.1371/journal.pone.0349297)
Supplement: S4 Table — (DOCX) [file pone.0349297.s011.docx]

**Supplementary Table S4** Results of Decomposition Analysis for HNC DALYs

| location | Overall difference | Aging | Population | Epidemiological change |
| --- | --- | --- | --- | --- |
| China | -165084 | 1298748.54  (786.72%) | 575573.1  (348.65%) | -2039406.13  (-1235.37%) |
| Democratic People's Republic of Korea | 20354.07 | 10173.3  (49.98%) | 8860.04  (43.53%) | 1320.73  (6.49%) |
| Japan | 118872 | 77705.5  (65.37%) | 4305.19  (3.62%) | 36861.3  (31.01%) |
| Mongolia | 919.47 | 927.22  (100.84%) | 1268.83  (138%) | -1276.58  (-138.84%) |
| Republic of Korea | 17692.2 | 41490.94  (234.52%) | 9661.08  (54.61%) | -33459.82  (-189.12%) |

DALYs, disability-adjusted life years.
